# Cholesteric Cellulose Liquid Crystal Fibers by Direct Drawing

**DOI:** 10.34133/research.0527

**Published:** 2024-11-07

**Authors:** Zhuohao Zhang, Qiao Wang, Yinuo Li, Chong Wang, Xinyuan Yang, Luoran Shang

**Affiliations:** ^1^ Shanghai Xuhui Central Hospital, Zhongshan-Xuhui Hospital, and the Shanghai Key Laboratory of Medical Epigenetics, the International Co-laboratory of Medical Epigenetics and Metabolism (Ministry of Science and Technology), Institutes of Biomedical Sciences, Fudan University, Shanghai 200032, China.; ^2^ Shanghai High School International Division, Shanghai 200231, China.

## Abstract

Polymer fibers are attracting increasing attention as a type of fundamental material for a wide range of products. However, to incorporate novel functionality, a crucial challenge is to simultaneously manipulate their structuring across multiple length scales. In this research, a facile and universal approach is proposed by directly drawing a pre-gel feedstock embedding a cellulose cholesteric liquid crystal (CLC). An in situ photo-polymerization process is applied, which not only allows for the continuous drawing of the filaments without breakup but also makes the final CLC fibers a colored appearance. More importantly, the multiscale properties of the fibers, such as their diameter, morphology, and the internal liquid crystalline ordering of the molecules (and thus structural color), can be manipulated by several controlling parameters. Combining this cross-scale tunability with a smart functional hydrogel system results in the formation of fibers with structural coloration, self-healing, electrical conduction, and thermal-sensing abilities. We believe that this platform can be extended to other hydrogel systems and will help unlock a wide variety of real-life applications.

## Introduction

Polymer fibers are widely used in a diverse range of products in daily life, and many functional fiber materials with excellent mechanical, optical, and responsive properties have been developed in recent years [1–5]. These materials have great potential for industrial applications in textiles, optoelectronics, energy generation, and healthcare [6–10]. Fibers can be fabricated using a variety of techniques. For example, surface-coating techniques can be used to modify or functionalize existing fibers, electrospinning can be used to produce nanofibers with a high surface area, printing techniques facilitate production of 3-dimensional (3D) structures, and thermal drawing supports fabrication of complex fiber electronics through simultaneous drawing of multiple materials [11–16]. Although some progress has been made, some limitations remain. For example, fiber properties are difficult to regulate simultaneously across different length scales, which includes the molecular interactions, organizational behavior of functional units, and macroscopic morphology [17–21]. The cross-scale properties of fibers are crucial for achieving unique functions. Therefore, novel techniques that consider the feedstock properties, ease of fiber processing, and simultaneous regulation of multiscale parameters is worth exploring.

**Fig. 1.**
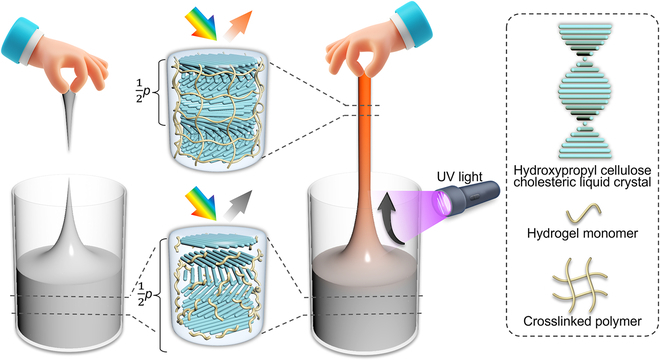
Schematic illustration of the direct-drawing technique for preparing cellulose CLC fibers with control over multiscale properties. Macroscopically, the diameter, cross-section shape, and structural color of the CLC fibers can be modulated by the nozzle size and shape, the drawing speed, the crosslinking position, etc. Microscopically, the CLC organization and the helical pitch can be modulated by the drawing speed and the crosslinking process.

This study presents a direct-drawing technique that can be used to control the molecular organization of the feedstock and the macroscopic size and morphology of the fibers simultaneously, as shown in Fig. 1. Hydroxypropyl cellulose (HPC) is a long-chain molecule derived from cellulose, which is inexpensive and has good biocompatibility [22,23]. It was used as the main component of the feedstock, which demonstrated a self-assembled cholesteric liquid crystal (CLC) state with a periodically aligned twisted-layer structure [24–29]. The HPC was combined with a pre-gel solution of a polymer, which resulted in a viscoelastic liquid that could be drawn directly using an arbitrary nozzle to form a filament. Homogeneous hydrogel CLC fibers were prepared continuously via in situ crosslinking of the filament before breakup. Remarkably, the drawing and crosslinking processes further regulated the CLC organization such that the CLC fibers generated structural colors from a colorless feedstock. The color could be controlled by varying the drawing speed and HPC content. Additionally, the diameter of the fibers could be precisely controlled by varying the drawing speed, HPC content, nozzle size, and crosslinking position. Furthermore, under appropriate crosslinking conditions, the fiber inherited the external morphology of the nozzle without capillary rounding, which allowed fibers with arbitrary shapes that deviated from the equilibrium to be obtained. Functional ingredients were incorporated into the fibers produced using this technique, and fibers with self-healing properties, good electrical conductivity, and thermal-sensing abilities were obtained without affecting the self-assembly of the CLC. Owing to the combination of structural coloration and functional properties, these fibers are suitable for a wide variety of applications. These results demonstrate that the proposed drawing method provides exceptional control over the material properties across multiple length scales, which represents a novel contribution to the field of functional CLC fibers. Moreover, this direct drawing holds universal applicability for diverse material systems, facilitating the development of CLC fibers with multiple functionalities for various applications such as optical devices, flexible electronics, intelligent textiles, and biosensors.

## Results

**Fig. 2.**
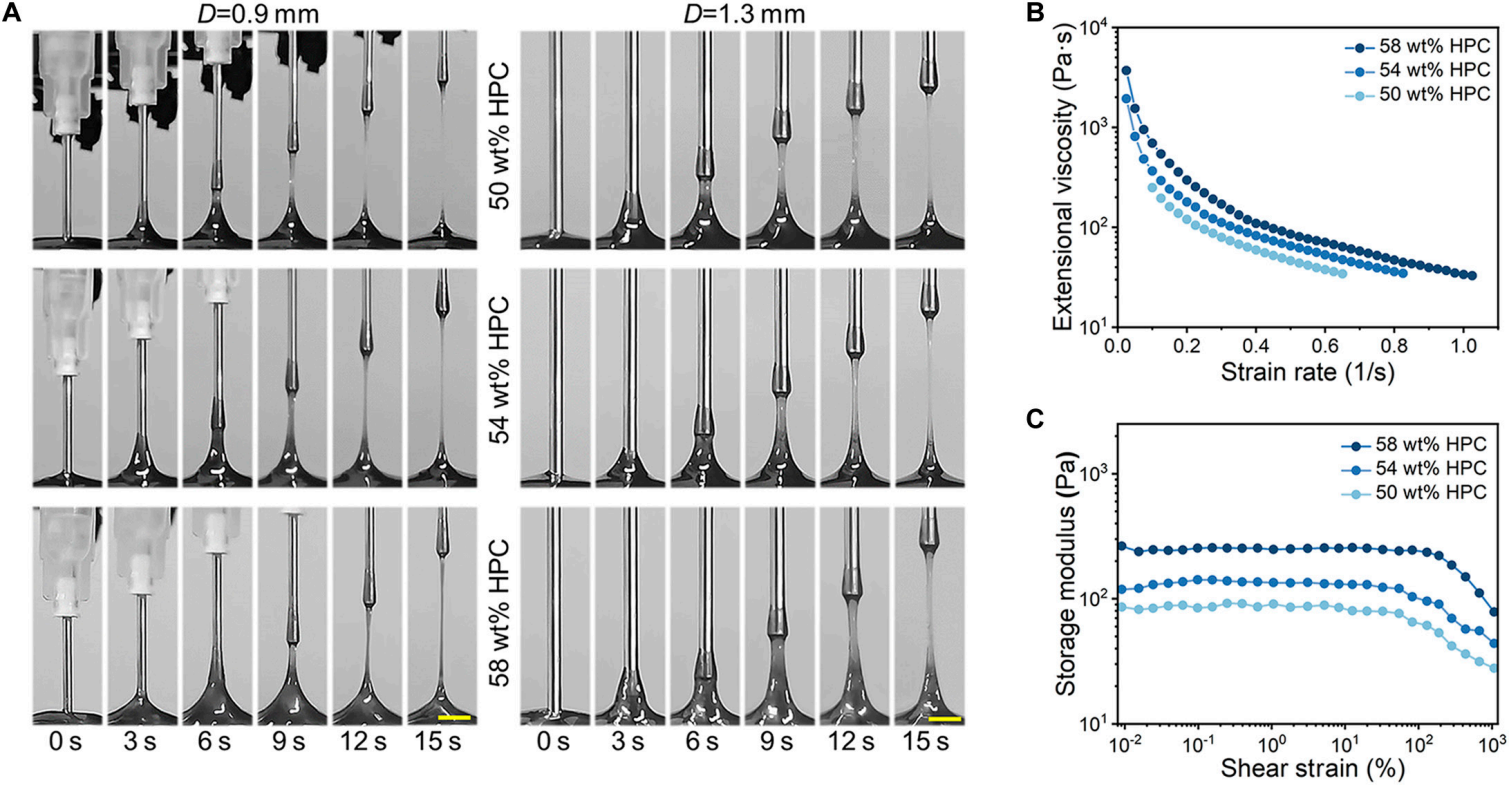
Photographs of the direct-drawing process and the rheological tests. (A) Photographs showing the feedstock drawing process with a constant drawing speed of 1 mm/s and nozzle diameters (*D*) of (left) 0.9 mm (right) and 1.3 mm. In each group, the HPC-PAM pre-gel solutions had HPC contents of (top) 50 wt%, (middle) 54 wt%, and (bottom) 58 wt%. Scale bars, 3 mm. (B) Graph showing the extensional viscosity as a function of the strain rate. (C) Rheological amplitude sweep tests on the feedstock samples with different HPC contents as the applied strain increased from 10^−2^% to 10^3^%. The angular frequency was constant at 10 rad/s.

In a typical experiment, HPC-based feedstocks were prepared by dispersing HPC (50 to 60 wt%) in a pre-gel polyacrylamide (PAM) solution (10 wt%). HPC is a long-chain cellulose derivative that is rich in hydroxyl groups. In concentrated aqueous solutions, the HPC molecules self-assemble via molecular interactions to form a CLC structure with periodically aligned twisted layers. In each layer, the HPC molecules are spontaneously arranged in parallel. That is, the material system exhibits inherent self-organization characteristics at the molecular level. A nozzle was used to draw the feedstock upward, and we studied its dynamic behavior. We introduced an automatic drawing setup with a cylindrical nozzle fixed on a *z*-axis lift perpendicular to the substrate where the feedstock was placed. The *z*-axis lift rose and fell at a constant speed and was controlled by an electric motor. A high-speed camera was used to record the entire drawing process, as shown in Fig. 2A. During the drawing process, the liquid interface rose to form a filament, which then underwent a necking process and eventually pinched off, leading to a breakup. At a constant drawing speed, we found that the pinch-off occurred later when the HPC content of the feedstock was higher (Fig. S1). Furthermore, at the same point in time, nozzles with a larger diameter (*D*) produced thicker filaments (Fig. S2). Notably, in a previous study, we also obtained filaments through continuous extrusion, which typically did not cause breakup [26]. Therefore, a detailed investigation of the breakup during the drawing process is required to determine a resolution.

The feedstock (i.e., the HPC-PAM pre-gel solution) was subjected to a series of rheological tests to investigate the drawing dynamics. Three samples with HPC contents of 58, 54, and 50 wt% were prepared with surface tensions of 31.0 ± 1.1 mN/m. External-viscosity tests indicated that the external viscosity of all three samples decreased as the strain rate increased, and the feedstock exhibited strain-thinning behavior, as shown in Fig. 2B. At the same strain rate, the sample with the highest HPC content exhibited the highest extensional viscosity, which increased its resistance to extension. Amplitude sweep tests were also performed, as shown in Fig. 2C, which showed that the samples had viscoelastic properties at applied strains between 0.01% and 100%. The storage modulus *G′* increased as the HPC content increased, which suggests that *G′* is dominated by the elastic contribution of the HPC. A frequency sweep test was performed at an applied strain of 0.01%. The loss factor (tan*δ*), which is equal to the ratio of loss modulus *G″* to *G′*, was plotted as a function of the angular frequency decreasing from 10^2^ to 10^−2^ rad/s, as shown in Fig. S3. The loss factor was greater than one for all the samples, which indicates that they exhibited typical viscoelastic liquid behavior.

**Fig. 3.**
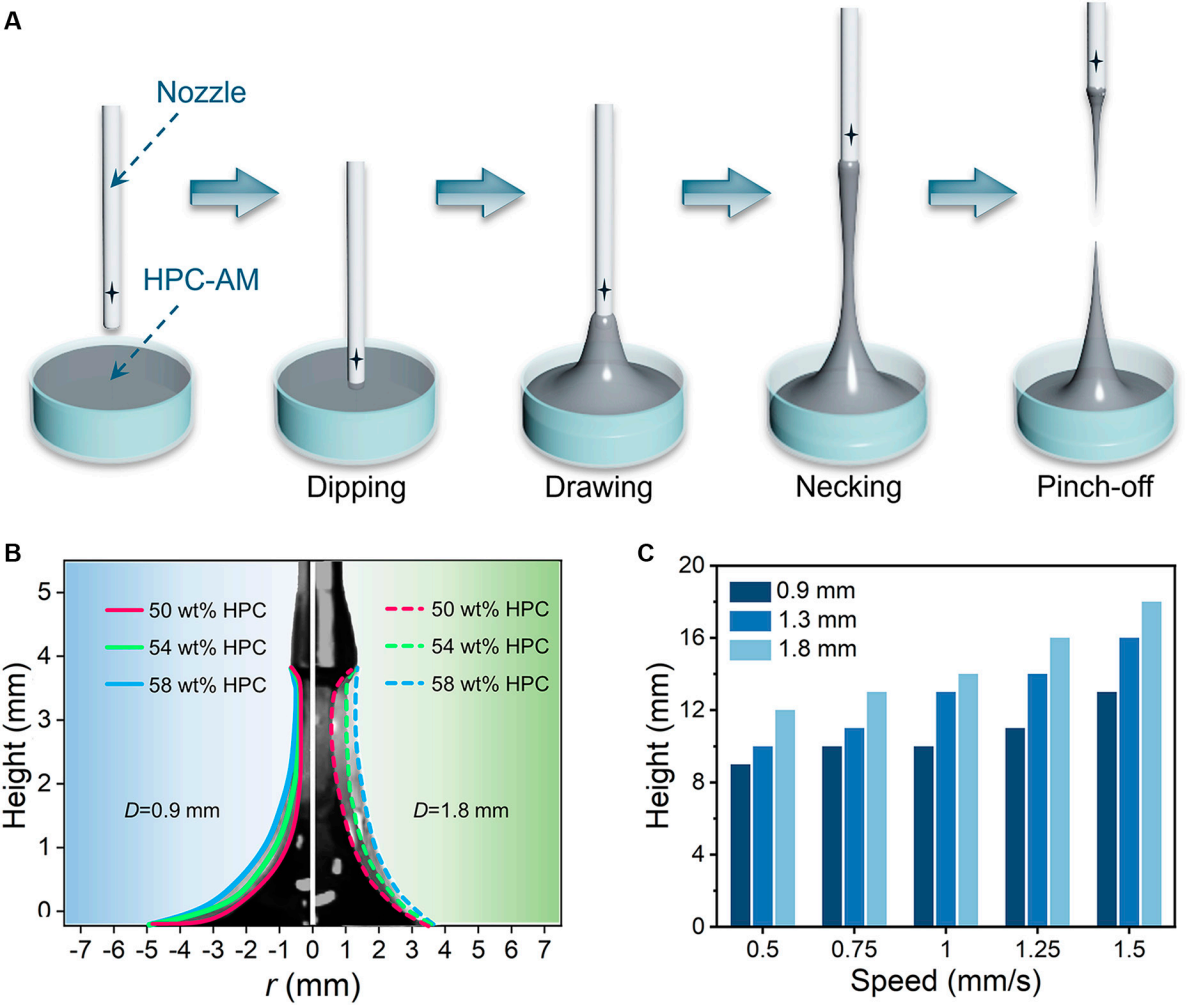
Analysis of the drawing dynamics of the liquid filament. (A) Schematic illustrations showing the dynamic behaviors of the liquid filaments during the drawing process, including stretching, necking, and pinch-off phenomena. (B) Profiles of the filaments drawn upward to the same height at a drawing speed of 1 mm/s using nozzles with diameters of 0.9 and 1.8 mm. (C) Breakup height of the sample with 54 wt% HPC as a function of the drawing speed and nozzle diameter.

A schematic of the necking and pinch-off mechanisms is shown in Fig. 3A. When the nozzle was dipped into the liquid and then lifted upward, a certain volume of the liquid was stretched out to form a filament. Given the viscoelastic liquid nature of the feedstock, the breakup of the filament may be attributed to ductile failure, which manifests as a necking and pinch-off process [30,31]. Therefore, we analyzed the factors affecting the breaking height of the filaments. The breaking height was defined as the distance between the nozzle and substrate when the minimum diameter of the filament was less than the minimum resolution of the camera. We compared the filament necks of the three samples drawn at the same height, as shown in Fig. 3B. The filament prepared using the sample with a high HPC content had a thicker neck, which indicated that the pinch-off phenomenon occurred later; hence, the breakup height was greater. This may be because the sample with a high HPC content had a higher extensional viscosity and viscoelasticity than the other samples, which had a stabilizing effect. Moreover, for a given sample, thicker nozzles drew a larger volume of liquid, which increased the breakup height, as shown in Fig. 3C. Furthermore, the dynamics of the liquid filament were also affected by the drawing speed. For the sample with 54 wt% HPC, the height of the breakup position increased as the drawing speed increased.

**Fig. 4.**
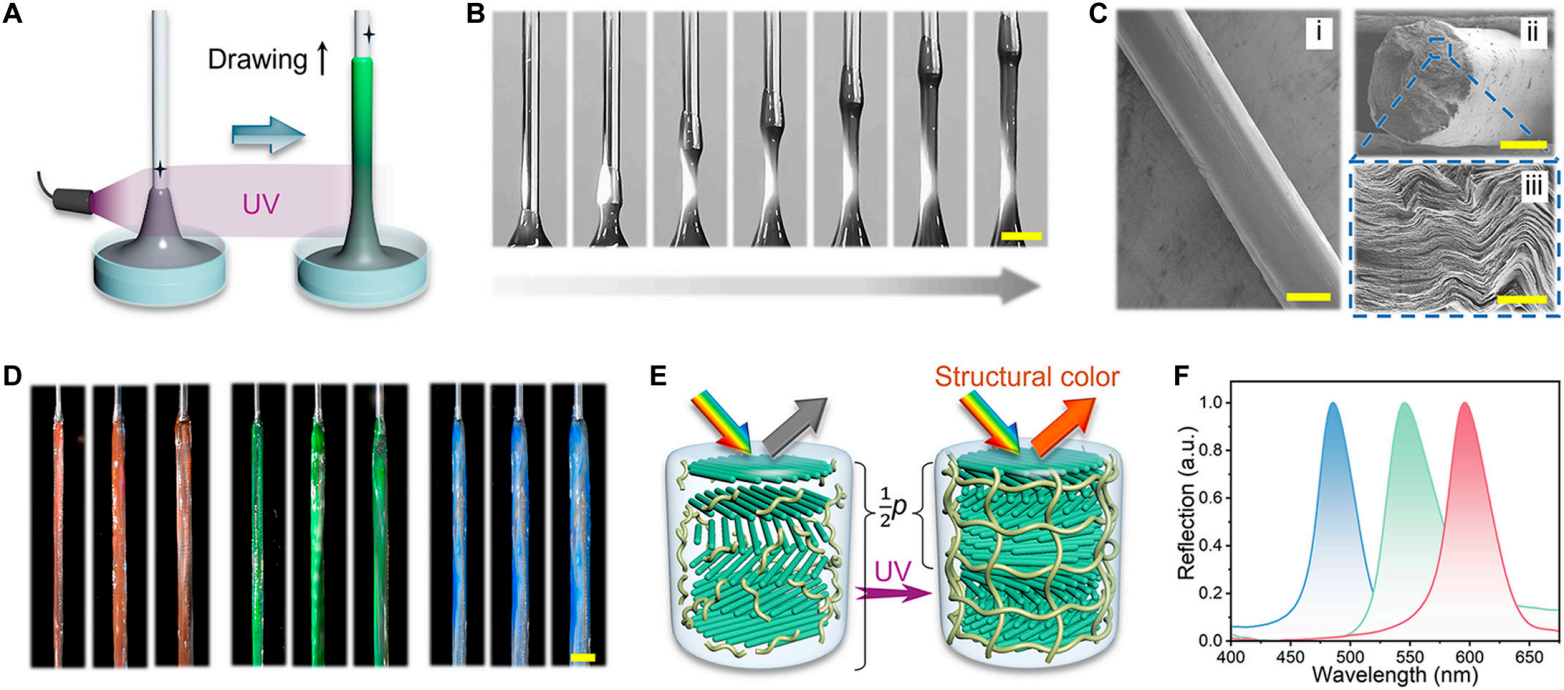
Continuous generation of structural-color CLC fibers by direct drawing and in situ photo-polymerization. (A) Schematic illustration and (B) photographs showing the fabrication of a single CLC fiber. Scale bar, 3 mm. (C) SEM images of the (i) side view of a CLC fiber, (ii) cross-section of a CLC fiber, and (iii) area indicated by the blue box in (ii). Scale bars, 500 μm (i and ii) and 5 μm (iii). (D) Photograph of CLC fibers with different visual colors. The HPC contents of the red, green, and blue fibers were 50, 54, and 58 wt%, respectively. Within each color group, the fibers were drawn using nozzles with diameters of (left to right) 0.9, 1.3, and 1.8 mm. Scale bar, 5 mm. (E) Schematic illustration showing the feedstock sample (reflection in the near-infrared region) and the fiber after direct drawing and in situ UV polymerization (reflection in the visible range). (F) Reflection spectra of the CLC fibers in (A). The HPC contents were (left to right) 58, 54, and 50 wt%.

The necking and pinch-off phenomena hindered the continuous generation of homogeneous fibers. Therefore, we introduced an in situ photo-polymerization strategy to solidify the filament before it was pinched off. As shown in Fig. 4A, a spot ultraviolet (UV) light source was positioned above the substrate and below the breakup height. Under the UV light, the acrylamide (AAm) monomers in the filament were crosslinked to form a polymer network as the drawing process proceeded continuously. Notably, although the filament was in a necking state (i.e., the diameter was not uniform) when the locally polymerized section was lifted, it assumed the role of the nozzle and drew the lower uncrosslinked section upward. Thus, HPC-PAM fibers with homogeneous diameters (0.5 to 3 mm) were generated continuously, as shown in Fig. 4B and Ci and Movie S1. The CLC fibers exhibited a Young's modulus of approximately 19 MPa (Fig. S4). Notably, the final length of the CLC fibers mainly depended on the maximum height of the *z*-axis lift and the volume of liquid used. In our tests, we used 3 ml of the aqueous HPC samples, which were drawn into CLC fibers that were approximately 20 cm long with homogeneous diameters.

The as-prepared CLC fibers exhibited metallic structural colors, as shown in Fig. 4D. The colors originated from the CLC organization of the HPC molecules, which indicates that the CLC mesophase was largely undisturbed by the drawing and crosslinking processes. Instead, UV crosslinking of the AAm monomers fixed the CLC structure, as shown in the scanning electron microscopy (SEM) images in Fig. 4Cii and iii. CLCs can be regarded as typical 1D photonic crystals with a characteristic photonic bandgap, and light with a wavelength within the bandgap is selectively reflected. In this study, the reflection peak position was determined by the helical pitch *p*, which is the distance covered by a 360° twist in the HPC molecular layers. When *p* was comparable to the wavelength of visible light, the CLC appeared as a structural color [27,32,33]. Remarkably, the HPC-PAM changed from a colorless liquid to a colorful hydrogel as the fibers were generated. This is probably because the CLCs in the PAM hydrogel were compressed during the UV crosslinking process, as shown in Fig. S5. Before crosslinking, the CLC twisted layers were expanded owing to the hydrogen bonds between HPC and AAm monomers; however, crosslinking broke these bonds and reduced the value of *p* [32]. Therefore, CLCs with reduced *p* values were fixed in the polymerized hydrogel networks, as shown in Fig. 4E. This process was recorded, and it is shown in Movie S1. This effect implies that the reflection peak and color of HPC-PAM fibers could be controlled by varying the HPC content. As the HPC concentration increased, *p* decreased and the structural color of the fibers shifted from red to blue, as shown in Fig. 4F and Fig. S6. Furthermore, doping with carbon black nanoparticles increased the color saturation.

**Fig. 5.**
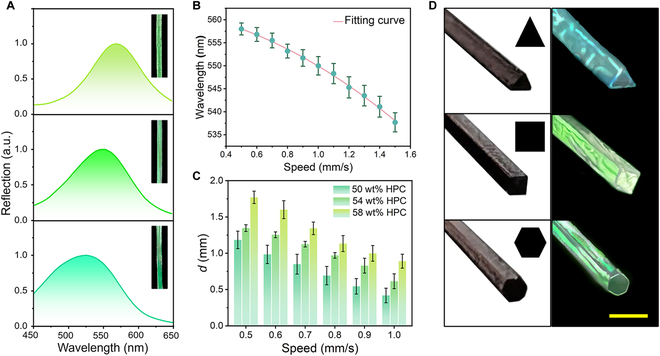
Structural modulation of the CLC fibers at multiple length scales. (A) CLC fibers with different reflection peaks and visual colors generated and prepared using 54 wt% HPC and drawing speeds of (top to bottom) 0.5, 1, and 1.5 mm/s. UV irradiation was conducted at the same position. (B) Correlation between the reflection peak position and drawing speed for HPC-PAM CLC fibers generated using a feedstock with an HPC content of 54 wt%. UV irradiation was applied at the same position. (C) Correlation between the fiber diameter (*d*) and drawing speed for HPC-PAM material systems with different HPC contents. UV irradiation was applied at a height of 1 cm, the nozzle diameter was 0.9 mm, and the drawing speed was 1 mm/s. (D) Structural colors of CLC fibers with triangular, square, and hexagonal cross-sections (right) and the corresponding nozzles used to produce them (left). Scale bar, 2 mm.

When all other conditions remained constant, the reflection peak of the prepared fibers became smaller as the drawing speed increased. This suggests that the drawing speed also affects the CLC organization behavior. Specifically, for the sample with 54 wt% HPC drawn at 0.5 to 1.5 mm/s, the reflection peak varied by up to 20 nm, as shown in Fig. 5B. Although the corresponding color change was not easily distinguished by the naked eye, such a correlation was observed in the reflection spectrum, as shown in Fig. 5A. This was determined by scratching a small piece of the HPC-PAM fiber longitudinally and characterizing the microstructure near the edge using SEM. A fibrillar texture was observed, as shown in Fig. S7, which was similar to the characteristic shear-induced texture reported elsewhere in the literature [34,35]. This suggests that, as the liquid filament was drawn upward, the polymer chains tended to reorient with the rotation of the helical axis [29,36]. This may tilt the alignment of the CLC domains and increase the incident angle *θ*. According to the De Vries equation, *λ* = *np*cos*θ* (where *λ* is the reflection wavelength and *n* is the average refractive index) [37], a reduction in *λ* causes a blueshift in the visual appearance and reflection peak. This drawing-induced alignment behavior may be used to precisely control the molecular organization and optical properties of HPC-PAM fibers.

The diameters of the fibers were also affected by the drawing speed. When the UV crosslinking position remained the same, the diameter of the fiber (*d*) decreased as the drawing speed increased, as shown in Fig. 5C. Furthermore, when the other conditions remained constant, the diameter of the fibers could also be modulated by other operational parameters such as the HPC content, nozzle diameter, and height of the UV light source, as shown in Figs. S8 and S9. This direct-drawing technique was further extended to generate heteromorphic fibers using nozzles with arbitrary shapes instead of circular ones. For example, triangular, square, and hexagonal fibers with vivid structural colors were prepared using nozzles with corresponding cross-sectional shapes (Fig. 5D). Notably, the fibers maintained the cross-sectional geometry of the nozzles without capillary rounding. This can be attributed to the high viscosity of the feedstock and rapid in situ crosslinking [38]. More precisely, the generation of these noncylindrical fibers was governed by the crosslinking kinetics, which were dependent on the intensity of the UV light and drawing speed (Fig. S10). When the intensity of the UV light was relatively low and the drawing speed was relatively high, the CLC fibers tended to revert to a circular cross-section. Thus, we expect that fibers with arbitrary geometries that deviate from the equilibrium may be prepared under appropriate conditions, which may be suitable for applications in novel anisotropic optical devices. Overall, the properties of the fibers were manipulated successfully, including the microscopic molecular organization and macroscopic size, morphology, and optical readout.

**Fig. 6.**
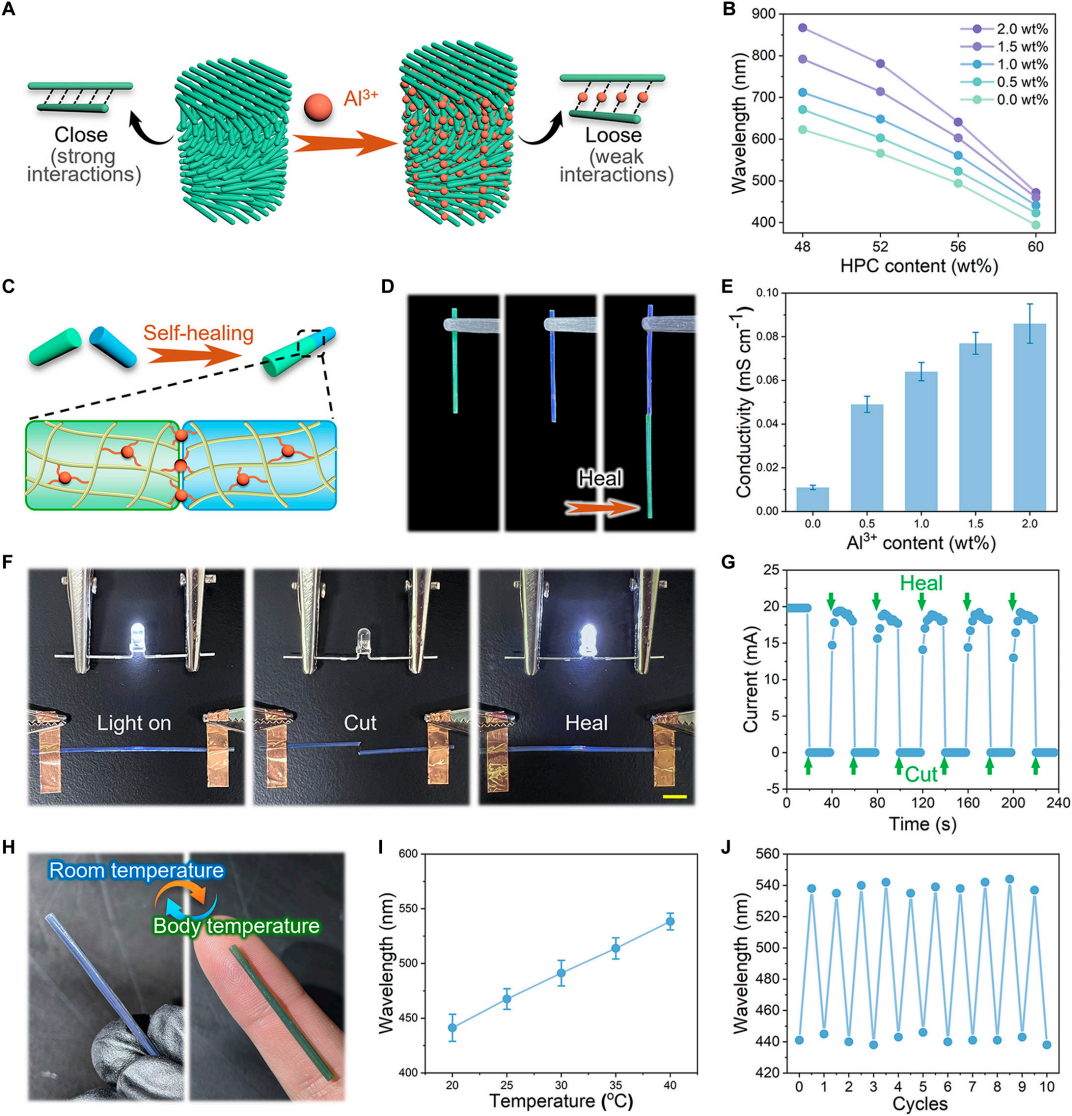
Multiple functionalities of the CLC fibers. (A) Schematic illustration of the color shift in HPC-PACA CLC fibers following the addition of Al^3+^. The Al^3+^ ions weaken the hydrogen bonds and other intermolecular interactions in the HPC molecular chain. (B) Reflection peak values for HPC-PACA CLC fibers with different HPC and Al^3+^ contents. (C) Schematic illustration of the self-healing mechanism of the CLC fiber caused by the coordination interactions formed between Al^3+^ and the free carboxyl group in the PACA hydrogel. (D) Self-healing performance of two different colored CLC fibers. (E) Conductivity of the HPC-PACA CLC fibers with different Al^3+^ contents. (F) Changes in the illuminance of an LED bulb during the cutting/healing process of an Al^3+^-doped HPC-PACA fiber that controlled the switching on/off of a circuit. Scale bar, 5 mm. (G) Current variations during the cutting/healing process of the Al^3+^-doped HPC-PACA fiber. (H) Variations in the appearance of an Al^3+^-doped HPC-PACA fiber at (left) room temperature (~25 °C) and (right) body temperature (~37 °C). (I) Reflection peak values of the HPC-PACA CLC fiber with an HPC content of 60 wt% at different temperatures. (J) Reflection peak variations of the HPC-PACA CLC fiber with an HPC content of 60 wt% cyclic temperature changes between 20 and 40 °C.

Compared to the previously reported extrusion-based technique [26], the technique presented in this study demonstrated superior cross-scale regulation ability. Therefore, we prepared multifunctional CLC fibers and explored their potential applications. We replaced the PAM hydrogel with a poly(acrylamide-co-acrylic acid) (PACA) hydrogel and incorporated Al^3+^. The rheological properties of the HPC-PACA hydrogels were similar to those of the HPC-PAM hydrogels, and they were suitable for the preparation of structurally colored CLC fibers via direct drawing, as shown in Fig. S11. The Al^3+^-doped HPC-PACA CLC fibers retained their structural color, which red-shifted in an Al^3+^-dependent manner. As a highly chaotropic ion, Al^3+^ can weaken the hydrophobic interactions of the alkyl groups and the hydrogen bonds of the hydroxyl groups in the HPC, which increases the helical pitch [39–41], as shown in Fig. 6A. Therefore, we investigated the effects of the Al^3+^ and HPC concentrations on the reflection peak position of the HPC-PACA CLC fibers and optimized the Al^3+^ (1 wt%) and HPC (50 to 60 wt%) concentrations to ensure that the reflection peaks of the HPC-PACA CLC fibers were in the visible range, as shown in Fig. 6B. Notably, Al^3+^ can also form metal-ion coordination interactions with the carboxyl groups in PACA [42–44]. The HPC-PACA CLC fibers exhibited self-healing, as shown in Fig. 6C. When two HPC-PACA fiber fragments came in contact, the strong coordination interactions and hydrogen bonds at the interface were sufficient for the fragments to form an integral fiber at room temperature (~20 °C) and withstand their own weight, as shown in Fig. 6D and Movie S2). This self-healing function can significantly extend the life of the CLC fibers and make them programmable for building higher-order constructs.

In addition to self-healing properties, the HPC-PACA CLC fibers with Al^3+^ exhibited excellent electrical conductivity, and there was a positive correlation between the Al^3+^ content and the electrical conductivity (Fig. 6E). A circuit was constructed using HPC-PACA fibers as the wires, and a light-emitting diode (LED) bulb was successfully illuminated when the circuit was powered on. Moreover, when the fiber was cut into two segments, the bulb illuminated immediately when contact between the two segments of the fiber was established owing to the self-healing properties, as shown in Fig. 6F. A cyclic cutting/healing test was conducted, and the real-time current was recorded, as shown in Fig. 6G. The current decreased to zero when the fiber was cut, and it returned to its initial value once the incision healed. The current in the circuit showed approximately no decay after repeated tests, which indicates that the self-healing ability had good repeatability and durability. The introduction of electrical conductivity into the structural-color CLC fibers significantly increases their potential applications in signal transmission, sensors, and smart wearable devices. Furthermore, the PACA hydrogel was thermo-responsive and the intermolecular hydrogen bonds broke as the temperature increased, which resulted in macroscopic expansion. This thermal expansion functioned synergistically with the thermosensitive properties of HPC. Thus, the CLC pitch of the HPC-PACA CLC fibers can be controlled by adjusting the temperature, which results in a dynamic color switch. As shown in Fig. 6H, the Al^3+^-doped HPC-PACA CLC fibers appeared blue at room temperature (~25 °C) and underwent a color transformation to green upon contact with the skin owing to body heat. This transition was accompanied by an upward shift in the position of the reflection peak and was reversible in the cyclic tests, as shown in Fig. 6I and J. HPC, the main component of the fibers, demonstrates good biocompatibility. Therefore, the structural color and aforementioned properties of the fibers make them suitable for use in intelligent fabrics, wearable devices, and electronic skin. Thus, the proposed direct-drawing technique can be used as a universal method for preparing novel fiber materials with various functions.

## Discussion

In summary, we proposed a direct-drawing technique for the generation of CLC fibers with multiscale structural regulation. A nozzle was dipped into a viscoelastic HPC-base pre-gel solution and used to stretch it out to form a filament, which was subject to necking and pinch-off phenomena. By combining this drawing technique with an in situ UV crosslinking strategy, CLC fibers with homogeneous diameters were generated continuously without breakup. The CLC mesophase formed by the self-assembly of HPC molecules was fixed in the crosslinked hydrogel network. This reflected visible light, which appeared as vivid structural colors. The diameter, shape, and helical pitch of the CLC fibers were finely modulated using various processing parameters including the nozzle diameter, nozzle shape, HPC content, height of the UV light source, and drawing speed. An Al^3+^-doped PACA hydrogel system was used to produce CLC fibers with self-healing properties, good electrical conductivity, and thermal-sensing functions without affecting the structural colors. Therefore, the proposed direct-drawing technique for the production of CLC fibers is a universal approach that can be used to design functional polymer CLC fibers with multiple functions. Moreover, the main component of the CLC fibers was HPC, which is a renewable material with excellent biocompatibility and has been used as a thickening agent in cosmetics and food. Therefore, the CLC fibers are expected to be suitable for applications in the biomedical and food industries. In future studies, we will endeavor to refine the material composition to improve the flexibility of the CLC fibers. This will enhance their suitability for application in textiles, including the emerging field of smart display fabrics. We also anticipate improvements in the uniformity of the CLC fibers and the development of large-scale manufacturing techniques using microfluidic spinning or industrial equipment. Furthermore, the viscoelasticity of the stock solution and breakup dynamics of the filament will be investigated systematically using advanced extensional rheology equipment. Overall, we believe that the proposed direct-drawing technique will guide the development of next-generation multifunctional polymer fibers.

## Materials and Methods

### Materials

Commercially available HPC, with a viscosity of 4.4 mPa·s when dispersed in a 2 wt% aqueous solution, was obtained from Nippon Soda Co. Ltd. AAm, *N*,*N*′-methylenebisacrylamide (MBA), 2-hydroxy-2-methylpropiophenone (HMPP), acrylic acid (AAc), aluminum chloride hexahydrate (AlCl_3_·6H_2_O), and carbon black were purchased from Sigma-Aldrich. Deionized water was obtained using a Milli-Q Plus 185 water purification system (Millipore).

### Preparation of HPC-PAM and HPC-PACA pre-gel solutions

Aqueous HPC-PAM solutions were prepared by dissolving 10 wt% AAm (monomer), 0.5 wt% MBA (crosslinking agent), 1 wt% HMPP (photo-initiator), 0.01 wt% carbon black, and 50 to 60 wt% HPC in deionized water. The solutions were stirred using a light-proof planetary mixer (Shengke Instruments) for a few days until homogeneous mixtures were obtained. Then, the samples were centrifuged at high speed (H3-18K, Kecheng Instruments; 20 °C, 9,000 rpm, 20 min) to remove any bubbles. The samples were stored away from light at 4 °C. Before use, the samples were removed from the refrigerator and centrifuged again. Aqueous HPC-PACA solutions were prepared by dissolving 5 wt% AAm, 5 wt% AAc, 2 wt% AlCl_3_·6H_2_O, 0.5 wt% MBA, 1 wt% HMPP, 0.01 wt% carbon black, and 50 to 60 wt% HPC in deionized water. The remaining preparation procedures were the same as those for the HPC-PAM solutions.

### Preparation of HPC CLC fibers

A sample of pre-gel solution, typically more than 2 ml, was placed on a glass substrate. A stainless-steel nozzle was fixed to the *z*-axis lift at an angle perpendicular to the glass substrate. The *z*-axis lift was used to control the nozzle, which was dipped into the solution and then driven at a constant speed to draw the solution upward. At a certain height above the liquid level, the pre-gel solution was photocrosslinked by irradiating it with a UV light source (UVP60, Runzhu Instrument) at a power density of 3,000 mW/cm^2^. Thus, CLC fibers with uniform diameters were obtained. CLC fibers with noncircular cross sections were fabricated using the same procedures with shaped nozzles prepared via 3D printing. The CLC fibers were characterized immediately after preparation; otherwise, the color underwent a blue shift owing to the gradual evaporation of water.

### Rheology tests

Extensional viscosity experiments were performed using a capillary break-up extensional rheometer (CaBER 1, Thermo Haake) with a 4-mm endplate, initial height of 2 mm, final height of 20 mm, and strike time of 60 ms at 25 °C. The dynamic filament diameters of the samples were recorded using a laser micrometer, which was part of the CaBER. Amplitude sweep tests were performed using a rotational rheometer (Discovery HR 10, TA instrument) with a 20 mm parallel plate geometry and a gap size of 1 mm at 20 °C. The applied strain was increased from 10^−2^% to 10^3^%, and the angular frequency was constant at 10 rad/s.

### Tests on the self-healing, electrical conductivity, and thermal-sensing properties

The self-healing properties of the HPC-PACA CLC fibers were investigated by placing two cut segments (generated from the 58 wt% HPC + 1 wt% Al^3+^ and 60 wt% HPC + 1 wt% Al^3+^ samples) at room temperature (~25 °C). The electrical conductivity was investigated by measuring the resistance of the HPC-PACA CLC fibers *R* (Ω) using a multimeter (DMM6500, Keithley Instrument) and calculating the conductivity of the samples *δ* (mS/cm) using the equation *δ = L*/(*R* × *S*), where *L* (cm) is the length of the HPC-PACA fiber and *S* (cm^2^) is the cross-sectional area of the fiber. In addition, on/off switching of the circuit was used to verify the self-healing properties and conductivity of the HPC-PACA CLC fiber (58 wt% HPC + 1 wt% Al^3+^) by showing the illumination on/off of a LED bulb (rated power = 0.06 W) before and after healing. Electric power was provided to the circuit using a digitally controlled direct current (DC)-stabilized voltage source (NPS01, Wanptek Instruments). During the cyclic cutting/healing tests, the current changes were recorded using a multimeter (DMM6500, Keithley Instrument). For the thermal-sensing tests, the HPC-PACA CLC fibers were heated to different temperatures using an electrical heater (MS-H-Pro, Dragon Lab Instrument).

### Characterization

Optical photographs were captured using a digital single-lens reflex camera (EOS R5, Canon) under white light with a black background. The surface tension was measured using a tensiometer (JCW-3600, Xiake). SEM images were captured using an SEM instrument (S-3000N, Hitachi). The stretchability and Young’s moduli of the CLC fibers were tested using a universal testing machine (XL-969S-500, Xianglong). The characteristic reflection spectra were measured using an optical microscope (CX33, OLYMPUS) equipped with a fiber-optic spectrometer (USB2000-FLG, Ocean Optics).

## Data Availability

Supporting information is available online.
