# Supplementary material for: Cholesteric Cellulose Liquid Crystal Fibers by Direct Drawing
Source: Research (Wash D C). 2024 Nov 7;7:0527. doi: 10.34133/research.0527 (PMC11541814; doi:10.34133/research.0527)
Supplement: Supplementary 1 — Figs. S1 to S11 Movies S1 and S2 [file research.0527.f1.zip › Supplementary Information.docx]

Supplementary Materials

**Movie S1**. Video showing the direct-drawing technique used to produce the HPC-based CLC fibers with structural colors generated at different drawing speeds. The HPC content of the feedstock was 54 wt%.

**Movie S2**. Video showing the self-healing properties of the HPC-PACA CLC fibers.

**
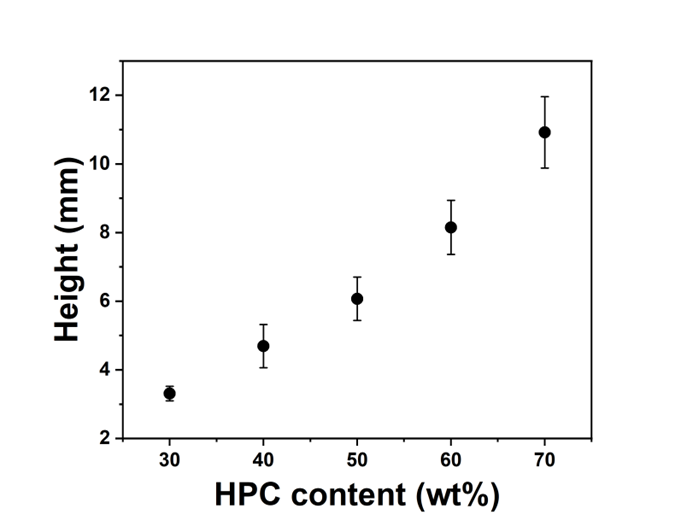
**

**Figure S1.** Breakup height of the liquid interface as a function of the HPC content. The nozzle diameter was 0.9 mm and the drawing speed was 1 mm/s.


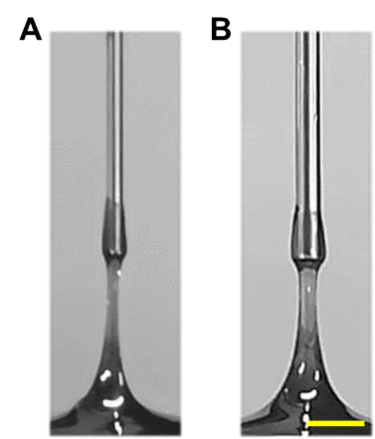


**Figure S2.** Photographs of the filaments drawn by nozzles with different diameters at the same time point. The nozzle diameters were (A) 0.9 mm and (B) 1.3 mm. In each case, the HPC content was 54 wt% and the drawing speed was 1 mm/s. The scale bar is 3 mm.


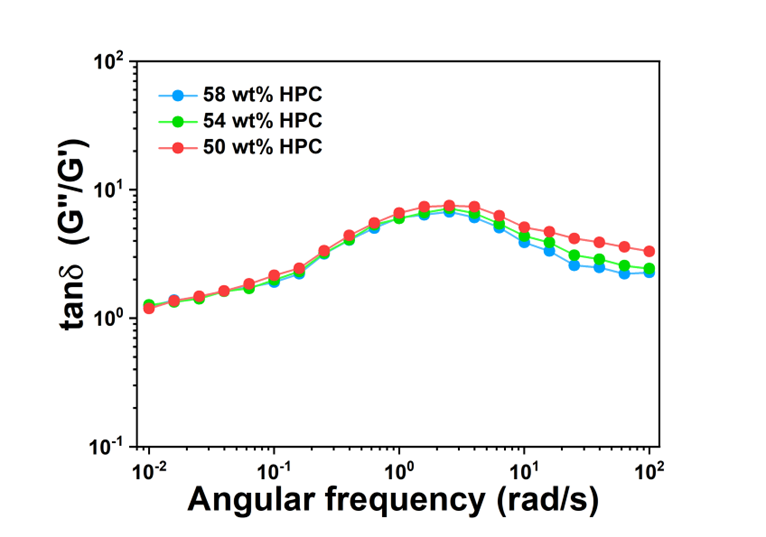


**Figure S3.** Rheological frequency sweep tests. The angular frequency was decreased from 10^2^ to 10^-2^, and tan*δ* was plotted at a constant applied strain of 0.1%.


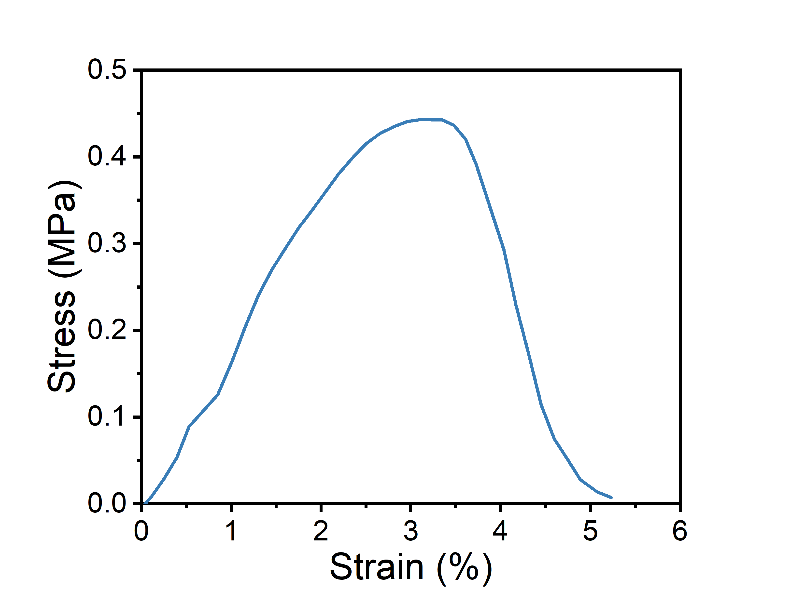


**Figure S4.** Stress–strain curve for a CLC fiber with an HPC content of 54 wt%.

**
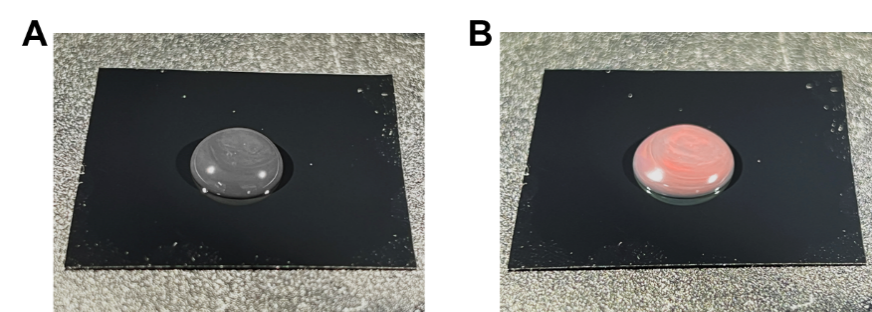
**

**Figure S5.** Photographs of bulk HPC-PAM (A) before and (B) after UV-crosslinking. Red-shift occurred owing to the compression of the CLCs. The HPC content was 50 wt%.

1.
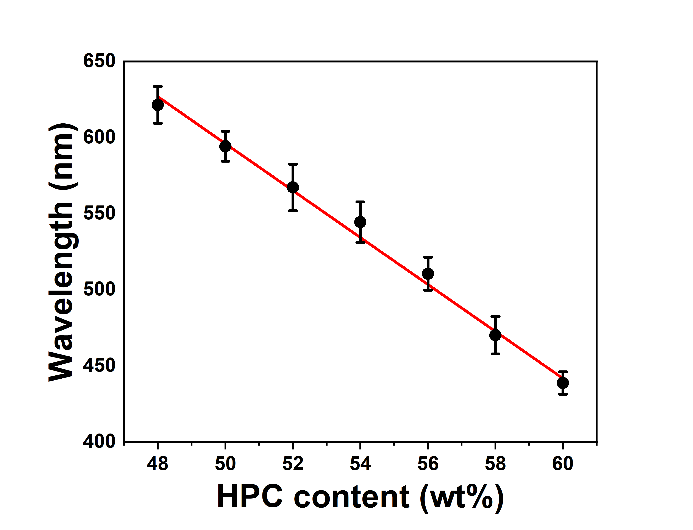


**Figure S6**. Reflection peak values of HPC-PAM CLC fibers as a function of the HPC content.


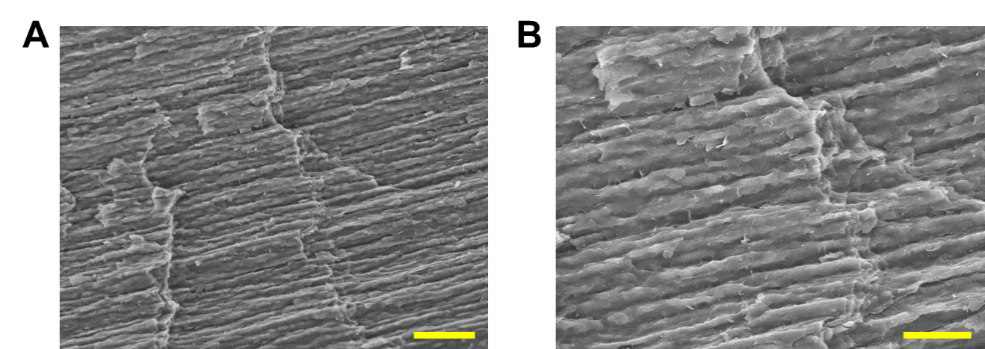


**Figure S7.** (A) An SEM image showing the microstructure of a small section near the edge of the HPC-PAM CLC fiber, which was scratched longitudinally. (B) A broader SEM image of the central region shown in (A). The scale bars are 20 μm in (A) and 10 μm in (B).


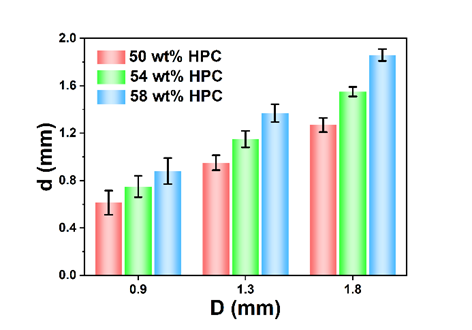


**Figure S8.** Diameters of the HPC-PAM CLC fibers with different HPC contents generated by nozzles with different diameters. In each case, the UV was applied at a height of 1 cm and the drawing speed was 1 mm/s.


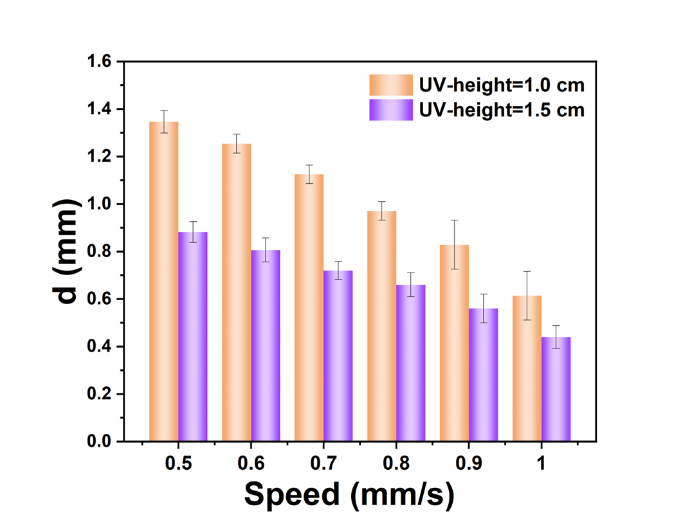


**Figure S9.** Diameter of the HPC-PAM CLC fibers produced using different drawing speeds. UV-height = 1.0 cm and UV-height = 1.5 cm indicate that the UV light source was placed 1.0 and 1.5 cm above the substrate, respectively.


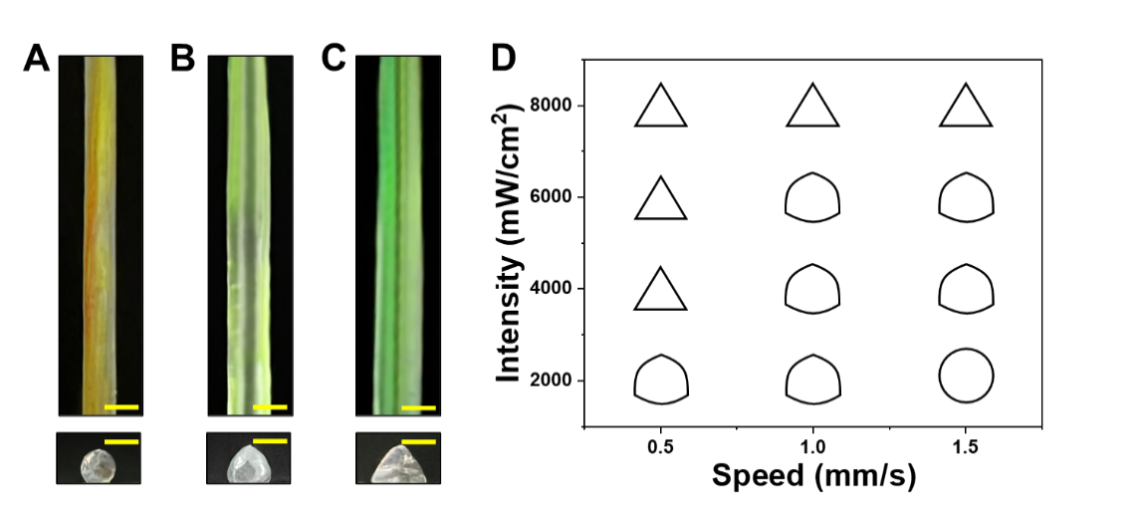


**Figure S10.** CLC fibers with different cross-sectional shapes prepared using nozzles with diameters of 1 mm. Representative images of (A) circular, (B) intermediate, and (C) triangular cross-sections. (D) Diagrams showing the cross-sectional shapes of the CLC fibers as functions of the UV-light intensity and drawing speed. In each case, the UV was applied at a height of 1 cm. The scale bars are all 1 mm.


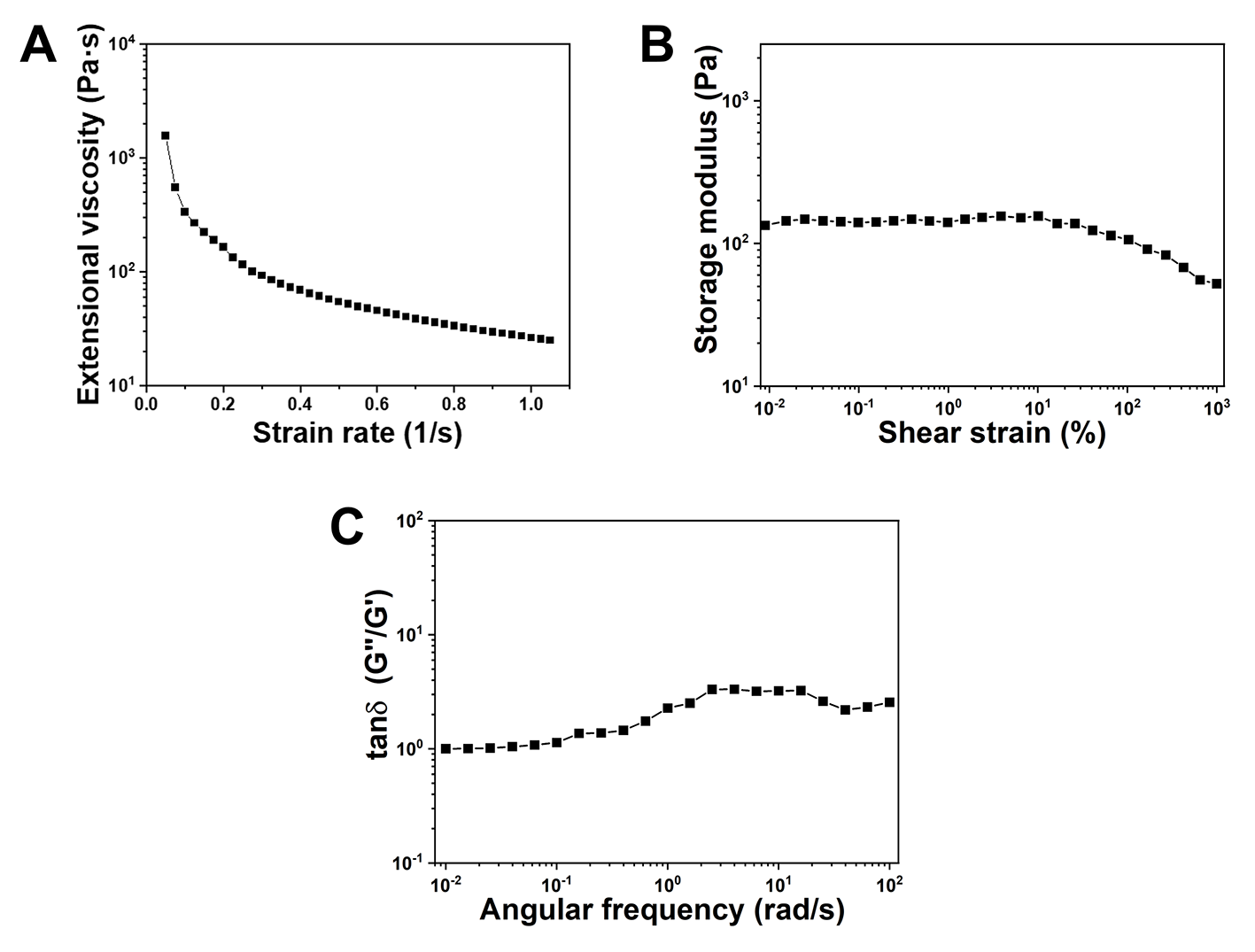


**Figure S11.** Rheological properties of the HPC-PACA feedstock sample with an HPC content of 50 wt%. (a) Graph showing the extensional viscosity as a function of the strain rate. (b) Rheological amplitude sweep test when the applied strain was increased from 10^-2^% to 10^3^%. The angular frequency was constant at 10 rad/s. (c) Rheological frequency sweep test when the angular frequency was decreased from 10^2^ to 10^-2^. tan*δ* was plotted at a constant applied strain of 0.1%.
